# Associations between fasting glucose rate-of-change and the missense variant, rs373863828, in an adult Samoan cohort

**DOI:** 10.1371/journal.pone.0302643

**Published:** 2024-06-03

**Authors:** Anna C. Rivara, Emily M. Russell, Jenna C. Carlson, Alysa Pomer, Take Naseri, Muagututia Seifuiva Reupena, Samantha L. Manna, Satupaitea Viali, Ryan L. Minster, Daniel E. Weeks, James P. DeLany, Erin E. Kershaw, Stephen T. McGarvey, Nicola L. Hawley

**Affiliations:** 1 Department of Chronic Disease Epidemiology, Yale School of Public Health, New Haven, Connecticut, United States of America; 2 Department of Human Genetics, School of Public Health, University of Pittsburgh, Pittsburgh, PA, United States of America; 3 Department of Biostatistics, School of Public Health, University of Pittsburgh, Pittsburgh, PA, United States of America; 4 Center of Surgery and Public Health, Brigham and Women’s Hospital, Boston, MA, United States of America; 5 Family Health Clinic, Apia, Samoa; 6 Naseri & Associates Health Consultancy Firm, Apia, Samoa; 7 Lutia I Puava Ae Mapu I Fagalele, Apia, Samoa; 8 Center for Craniofacial and Dental Genetics, Department of Oral and Craniofacial Sciences, School of Dental Medicine, University of Pittsburgh, Pittsburgh, PA, United States of America; 9 Oceania University of Medicine, Apia, Samoa; 10 Advent Health Orlando, Translational Research Institute, Orlando, FL, United States of America; 11 Division of Endocrinology, Department of Medicine, University of Pittsburgh, Pittsburgh, PA, United States of America; 12 Department of Epidemiology, International Health Institute, School of Public Health, Brown University, Providence, RI, United States of America; 13 Department of Anthropology, Brown University, Providence, RI, United States of America; Centre Hospitalier Sud Francilien, FRANCE

## Abstract

**Background:**

The A allele of rs373863828 in CREB3 regulatory factor is associated with high Body Mass Index, but lower odds of type 2 diabetes. These associations have been replicated elsewhere, but to date all studies have been cross-sectional. Our aims were (1) to describe the development of type 2 diabetes and change in fasting glucose between 2010 and 2018 among a longitudinal cohort of adult Samoans without type 2 diabetes or who were not using diabetes medications at baseline, and (2) to examine associations between fasting glucose rate-of-change (mmol/L per year) and the A allele of rs373863828.

**Methods:**

We describe and test differences in fasting glucose, the development of type 2 diabetes, body mass index, age, smoking status, physical activity, urbanicity of residence, and household asset scores between 2010 and 2018 among a cohort of n = 401 adult Samoans, selected to have a ~2:2:1 ratio of GG:AG: AA rs373863828 genotypes. Multivariate linear regression was used to test whether fasting glucose rate-of-change was associated with rs373863828 genotype, and other baseline variables.

**Results:**

By 2018, fasting glucose and BMI significantly increased among all genotype groups, and a substantial portion of the sample developed type 2 diabetes mellitus. The A allele was associated with a lower fasting glucose rate-of-change (β = −0.05 mmol/L/year per allele, *p =* 0.058 among women; β = −0.004 mmol/L/year per allele, *p =* 0.863 among men), after accounting for baseline variables. Mean fasting glucose and mean BMI increased over an eight-year period and a substantial number of individuals developed type 2 diabetes by 2018. However, fasting glucose rate-of-change, and type 2 diabetes development was lower among females with AG and AA genotypes.

**Conclusions:**

Further research is needed to understand the effect of the A allele on fasting glucose and type 2 diabetes development. Based on our observations that other risk factors increased over time, we advocate for the continued promotion for diabetes prevention and treatment programming, and the reduction of modifiable risk factors, in this setting.

## Introduction

Type 2 diabetes mellitus has quickly become pervasive globally, with an estimated 537 million people impacted as of 2021 [1]. Pacific Islanders experience a disproportionately high prevalence of type 2 diabetes mellitus compared to other settings globally, with the Western Pacific Region (including Samoa) accounting for 38% of the global prevalence among adults [1]. Concomitant with the high prevalence of type 2 diabetes mellitus, Pacific Islanders also have comparatively high fasting glucose (FG) levels. Between 1980 and 2008, average FG levels in Oceania increased by 0.22 mmol/L per decade among men and 0.32 mmol/L per decade among women compared to global average per decade increases of 0.07 mmol/L among men and 0.09 mmol/L among women [2, 3]. The rapid increase in type 2 diabetes mellitus in Pacific Islander populations has largely been ascribed to changes in lifestyle and diet, and corresponding increases in overweight and obesity prevalence over the past several decades [3].

The high prevalence of type 2 diabetes mellitus among Pacific Islander populations is exemplified in Samoa, an independent Polynesian island nation with a population of approximately 203,774 [4]. In 2013 (the most recent available national data), the prevalence of type 2 diabetes mellitus in Samoa was 19.6% among adult men (having increased from 1.2% in 1978) and 19.5% among adult women (from 2.2% in 1978) [5]. A leading risk factor for type 2 diabetes mellitus, obesity prevalence [Body Mass Index (BMI) ≥ 30 kg/m^2^] was also found to have increased from 27.7% to 53.1% among adult men, and 44.4% to 76.7% among adult women between 1978 and 2013 [5].

The identification of a missense variant (rs373863828, c.1370G>A, p.R457Q) in the CREB3 Regulatory Factor (*CREBRF*), which occurs at a relatively high frequency within Pacific Islander populations (minor allele frequency [MAF] range: 0.042–0.259) [6–14], suggests that genetic influences might mitigate type 2 diabetes mellitus risk for some in this population and may attenuate the overall population prevalence. In a genome-wide association study (GWAS) of adult Samoans (*n* = 3,072) [6], the minor (A) allele of rs373863828 was paradoxically associated with greater BMI but lower odds of type 2 diabetes mellitus, and significantly lower FG among people without type 2 diabetes mellitus (with an effect size of 0.09 mmol/L lower FG for each copy of the minor allele) [6]. The same protective association between the A allele of rs373863828 and odds of type 2 diabetes mellitus has been replicated in Pacific Islanders living in Saipan and Guam [7], Native Hawaiians [8], and Māori and Pacific people living in New Zealand [13]. However, because these studies have been cross-sectional, the extent to which rs373863828 is associated with change in FG over time remains unknown.

Here, we present the first assessment of associations between rs373863828 genotype and FG rate-of-change, in mmol/L per year, in a sample of adult Samoans residing on ’Upolu Island in the independent nation of Samoa. This paper reports (1) changes in FG, and the development of type 2 diabetes mellitus over a 7–9-year period, and (2) associations between rs373863828 genotype and FG rate-of-change.

## Materials and methods

### Study design and study population

The *Soifua Manuia* (‘Good Health’ in Samoan) study was conducted between August 1, 2017, and February 28, 2019 (hereafter referred to as 2018 for brevity) and followed up n = 519 participants who previously took part in a genome-wide association study (GWAS) in 2010 (February to July). The country is divided into four census regions (ordered from most urban to rural): [15] the Apia Urban Area (AUA; Apia is the capital city), Northwest ’Upolu (NWU), the Rest of ’Upolu (ROU), and Savai’i (SAV). In 2010, the GWAS was conducted among Samoan adults from 33 villages across all four census regions on the islands of ‘Upolu and Savai’i [6]. The 2010 protocol included the collection of anthropometric, biochemical, and questionnaire data from *n* = 3,475 eligible individuals between 24.5 and 65 years old. [16] Detailed descriptions of the study protocol and methods, eligibility, and sampling strategy are described elsewhere [16].

A sample of *n* = 519 men and women from the 2010 GWAS cohort were re-recruited to participate in the 2018 follow-up study. The follow-up study aimed to characterize associations between rs373863828 (identified in the 2010 GWAS) and metabolic and behavioral traits influencing energy homeostasis [17]. Recruitment details in more depth can be found elsewhere [17]. Participants were initially recruited purposively by genotype (for each participant homozygous for the rs373863828 minor allele [AA], two heterozygous participants [AG] and two participants homozygous for the major allele [GG] were recruited) with attempts to match for age (± 5 years), sex, and census region. Due to unforeseen difficulties with matching, the sampling strategy changed mid-protocol to prioritize AA and AG individuals without explicitly matching for age, sex, and census region [17]. Participants were between the ages of 32 and 72 years old, resided in the three census regions on the island of ‘Upolu, had no physical or cognitive disabilities that prevented their completion of the study protocol, and were not currently pregnant. Additionally, participants could not have had weight loss surgery, be taking weight loss medications, or have experienced > 5% loss of their body weight over the past 12 months. We did not recruit individuals from the Savai’i census region during the 2018 follow-up as the intensive protocol could not be logistically completed in that more rural census region.

### Informed consent and ethical review

The 2010 data collection was approved by Brown University’s Institutional Review Board and the Health Research Committee of the Samoan Ministry of Health. The 2018 study was approved by Yale University’s and Brown University’s Institutional Review Boards (Yale served as the IRB of record, IRB#1604017547), and the Health Research Committee of the Samoan Ministry of Health. All participants provided their written informed consent, consenting separately at each time point.

### Outcomes: Fasting glucose and type 2 diabetes mellitus development

In 2010, venous blood samples were collected in 10 mL serum separator vacutainers and centrifuged on site. Samples were stored at −40°C before being shipped to the Northwest Lipids Labs, Seattle, WA, for FG assay on a Hitachi 917 Clinical Chemistry auto-analyzer [14]. In 2018, venous blood samples were collected in 10 mL sodium fluoride vacutainers, centrifuged on site, and stored at −80°C. Samples were shipped to the University of Pittsburgh for assay. Plasma glucose levels were determined using an Analox GM9 Glucose Analyzer. At both timepoints, participants were classified as having diabetes if they reported current use of diabetes medication (participants were asked if they used pills or insulin for their diabetes; subsequently they were asked to name the medications. The types of diabetes-specific medications available in 2010 and 2018 were limited with most participants excluded for this criterion stating they used metformin, sulfonylureas, or insulin). Additionally, participants were excluded if they had had FG ≥ 7 mmol/L [18].

### Covariates and background characteristics

Questionnaire data from the 2010 study used here included: demographic characteristics (age, sex, census region), health history (self-report of diabetes based on receiving a prior diagnosis from a physician and medication use), household socioeconomic position (measured using a twelve-item household assets inventory), current cigarette smoking [19], and a dichotomous measure of whether participants meet WHO weekly recommendations for physical activity (assessed via the WHO Global Physical Activity Questionnaire [GPAQ]) [20]. An additive household asset score was calculated based on ownership of the following twelve items: refrigerator, freezer, portable stereo, TV, VCR, couch, washing machine, phone, car, house, plumbing and stove [21]. GPAQ responses were used to determine whether participants met the WHO-recommended 600 metabolic equivalent minutes (approximately 150 minutes) of moderate-to-vigorous physical activity (MVPA) per week [20].

Anthropometric measures (weight and height) were collected in duplicate and averaged for analyses: weight was collected to the nearest 0.1 kg (Tanita HD 351, Tanita Corporation of America, IL), and height to the nearest 0.1 cm using a portable stadiometer (GPM, Pfister Imports, NY). BMI was calculated as kg/m^2^ and was used as the indicator of body size in the analyses. BMI rate-of-change between 2010 and 2018 was calculated as the difference in BMI between 2010 and 2018, divided by the time in years between assessments.

Questionnaire and anthropometric data collection in 2018 mirrored protocols used in 2010 [17].

### Genotype

Consistent with prior studies, rs373863828 genotype was modeled additively, representing the number of A alleles an individual carries (i.e., GG = 0, AG = 1, AA = 2).

### Data analyses

All statistical analyses were completed using R version 3.6.0 [22]. Models for fasting glucose were fit using the lm function in R; visualizations were created with the ggplot2 package [23].

### Descriptive statistics

Descriptive statistics were calculated for all analyzed variables. Differences in participant characteristics between timepoints were tested with Student’s paired *t* tests (continuous) and McNemar’s tests of association (categorical). Differences in participant characteristics by sex were tested with Welch’s unpaired *t* test. Confidence intervals calculated using the exact method of the Poisson distribution were provided to assess the average annual number of new cases of type 2 diabetes mellitus between 2010 and 2018.

### FG rate-of-change

FG rate-of-change between 2010 and 2018 was calculated as the difference in FG between 2010 and 2018, divided by the time in years between assessments. Linear regression models with FG rate-of-change (mmol/L per year) as the outcome were fit in the full cohort and in sex-stratified analyses. In addition to including BMI rate-of-change (calculated similarly to FG rate-of-change) as a covariate, we also included 2010 measures of age, age^2^ (to model the non-linear effect of age), BMI (kg/m^2^), FG (mmol/L), rs373863828 genotype, census region of residence (AUA, NWU, or ROU; a proxy for urbanicity and exposure to nutrition transition, reference level [ref]: AUA), physical activity as a dichotomous measure of whether participants met WHO guidelines (ref: no), household asset scores, and current smoking status (ref: no) as covariates. For individuals with missing values for ≤ 2 items in the household assets inventory in 2010 or 2018 (*n* = 5 and *n* = 13, respectively), the missing values were imputed with the *bnstruct* R package using ten nearest neighbors [24]. Sex (ref: male) was included as a covariate in the non–sex-stratified analyses.

## Results

**Fig 1:**
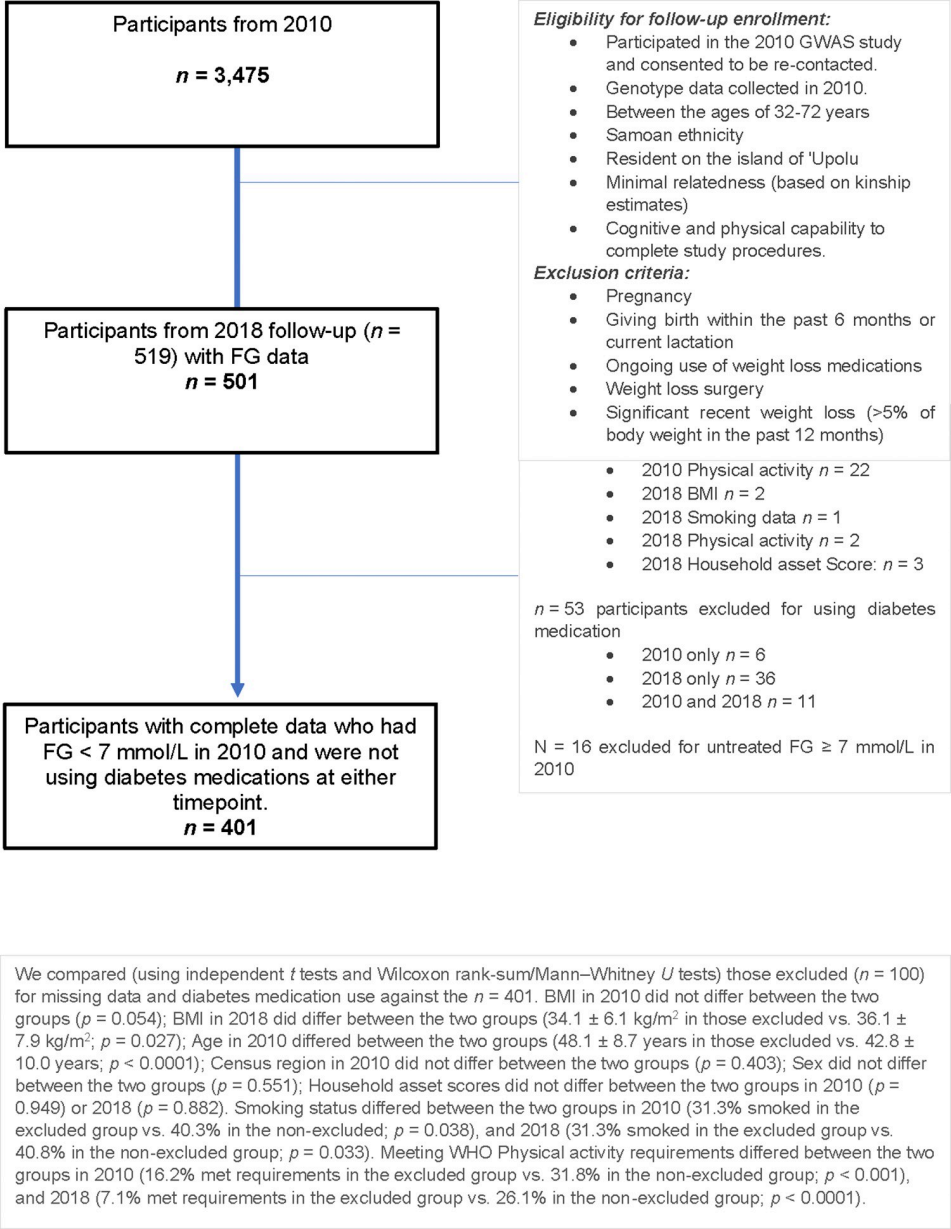
Flow diagram of included participants.

Of the *n* = 519 GWAS participants followed up in 2018, *n* = 501 participants had FG measures from 2018 (Fig 1). We excluded *n* = 31 participants who were missing covariate data at either timepoint. Next, we chose to exclude 53 individuals who reported using diabetes medication in either 2010 or 2018 from the analyses. The choice to exclude was made because the most frequently reported diabetes medications in the sample act to acutely lower blood glucose levels either directly or through influencing insulin secretion [25, 26]. Finally, we chose to exclude an additional sixteen individuals who had FG ≥ 7 mmol/L (indicative of diabetes) [18] in 2010, who were not on medication or excluded previously for missing data. This choice was motivated by evidence demonstrating that hyperglycemia and diabetes duration may impact glucose metabolism and beta cell function [27–29], therefore, their inclusion would likely bias our analyses of FG rate-of-change. Our analyses were subsequently based on *n* = 401 participants (Fig 1). Differences between those excluded from the analyses based on missing data and/or diabetes medication use are noted in Fig 1. Of note, those included were significantly younger than the excluded group and mean BMI in 2018 was higher among those included.

### Sample characteristics, development of type 2 diabetes mellitus, and mean FG levels between 2010 and 2018

Table 1 shows participant characteristics in 2010 and 2018 by sex. At baseline in 2010, none of the included participants had type 2 diabetes mellitus; by 2018, n = 38 (20.2%) of men and n = 33 (15.5%) of women developed type 2 diabetes mellitus. As expected from the number of individuals who developed type 2 diabetes mellitus in both sexes, increases in sample mean FG levels were observed between 2010 and 2018. Mean FG among the total sample increased 1.5 ± 2.2 mmol/L between 2010 and 2018 (1.4 ± 2.0 mmol/L among men, 1.6 ± 2.3 mmol/L among women; *p* = 0.57). Between 2010 and 2018, BMI and household assets scores increased among the sample. Mean BMI increased in the total sample 2.2 ± 3.6 kg/m^2^ between 2010 and 2018, but the average change in BMI differed between men and women (1.7 ± 3.4 kg/m^2^ among men, 2.7 ± 3.6 kg/m^2^ among women; *p* = 0.003). Compared to 2010, both men and women owned an average of one more consumer durable item in 2018, increasing their household asset score by one point. Among women only, the proportion of participants meeting physical activity recommendations was lower in 2018 than in 2010 (15.0% vs. 24.9%, *p* = 0.012).

**Table 1:** Descriptive statistics of n = 401 adult Samoans in 2010 and 2018.

Combined (*n* = 401)				Men (*n* = 188)			Women (*n* = 213)		
	2010	2018	*p* values	2010	2018	*p* values	2010	2018	*p* values
Age (years)	42.9 (10.1)	51.1 (10.1)	-	44.9 (10.4)	53.2 (10.4)	-	41.1 (9.4)	49.3 (9.5)	-
BMI (kg/m^2^)	33.9 (6.7)	36.1 (8.0)	6.3 × 10^−31^	31.7 (5.9)	33.4 (7.1)	2.5 × 10^−10^	35.8 (6.8)	38.6 (7.9)	6.9 × 10^−23^
BMI change (kg/m^2^)^a^	2.2 (3.6)			1.7 (3.4)			2.7 (3.6)		
BMI rate-of-change ((kg/m^2^)/year)	0.27 (0.43)			0.20 (0.41)			0.34 (0.44)		
Current smoker (%)	162 (40.4%)	163 (40.6%)	1.000	104 (55.3%)	102 (54.3%)	0.868	58 (27.2%)	61 (28.6%)	0.700
Meets PA recs (%)	128 (31.9%)	105 (26.2%)	0.073	75 (39.9%)	73 (38.8%)	0.915	53 (24.9%)	32 (15.0%)	0.012
Household asset scores	5.6 (2.8)	6.6 (2.9)	2.1 × 10^−10^	5.8 (2.8)	6.7 (3.0)	1.8 × 10^−5^	5.5 (2.8)	6.5 (2.8)	3.0 × 10^−6^
***Metabolic phenotypes***									
Type 2 diabetes (%)^b^^c^	0	71 (17.7%)	9.8 × 10^−17^	0	38 (20.2%)	1.9 × 10^−9^	0	33 (15.5%)	2.5 × 10^−8^
Average type 2 diabetes incidence/year ^c^	0	8.9	CI: 6.9–11.2	0	4.8	CI: 3.4–6.5	0	4.1	CI: 2.8–5.8
Fasting glucose (mmol/L)	4.8 (0.8)	6.3 (2.3)	3.1 × 10^−36^	4.9 (0.8)	6.3 (2.1)	8.9 × 10^−19^	4.7 (0.8)	6.2 (2.4)	3.0 × 10^−19^
Fasting glucose change (mmol/L) 2010–2018^d^	1.5 (2.2)			1.4 (2.0)			1.6 (2.3)		
Fasting glucose rate-of-change ((mmol/L)/year) ^e^	0.19 (0.27)			0.18 (0.25)			0.19 (0.29)		
***rs373863828 genotype***									
GG (%)	162 (40.4%)	-	-	77 (41.0%)	-	-	85 (39.9%)	-	-
AG (%)	162 (40.4%)	-	-	76 (40.4%)	-	-	86 (40.4%)	-	-
AA (%)	77 (19.2%)	-	-	35 (18.6%)	-	-	42 (19.7%)	-	-
***Census region***									
Apia Urban Area (AUA) (%)	85 (21.2%)	88 (21.9%)	0.505	40 (21.3%)	43 (22.9%)	0.371	45 (21.1%)	45 (21.1%)	1.000
Northwest ‘Upolu (NWU) (%)	169 (42.1%)	164 (40.9%)	0.228	78 (41.5%)	75 (39.9%)	0.371	91 (42.7%)	89 (41.8%)	0.683
Rest of ‘Upolu (ROU) (%)	147 (36.7%)	149 (37.2%)	0.724	70 (37.2%)	70 (37.2%)	1.000	77 (36.2%)	79 (37.1%)	0.683

mean (SD) or *n* (%)

*p* values indicate change between 2010 and 2018 unless otherwise noted; *p* values calculated with Student’s paired *t* test for continuous traits and McNemar’s test for binary traits

^a^ Difference in BMI change by Sex (*p* = 0.003)

Meets PA recs: meets WHO recommendations for weekly MET minutes.

2010: excluded individuals with FG ≥ 7 mmol/L or diabetes medication use; 2018: excluded individuals with diabetes medication use, T2D determined by FG ≥ 7 mmol/L

^b^ Difference in type 2 diabetes development by sex (*p* = 0.22)

^c^ 2010 Type 2 diabetes prevalence and annual incidence of 0 is due to the selective sampling strategy of excluding those with type 2 diabetes.

CI indicates the 95% confidence interval for the mean incidence per year of the Poisson distribution using the exact method.

^d^ *p* value for difference in values between men and women calculated with Welch’s unpaired *t* test, *p* = 0.57

^e^*p* value for difference in values between men and women calculated with Welch’s unpaired *t* test; *p* = 0.54

**Fig 2:**
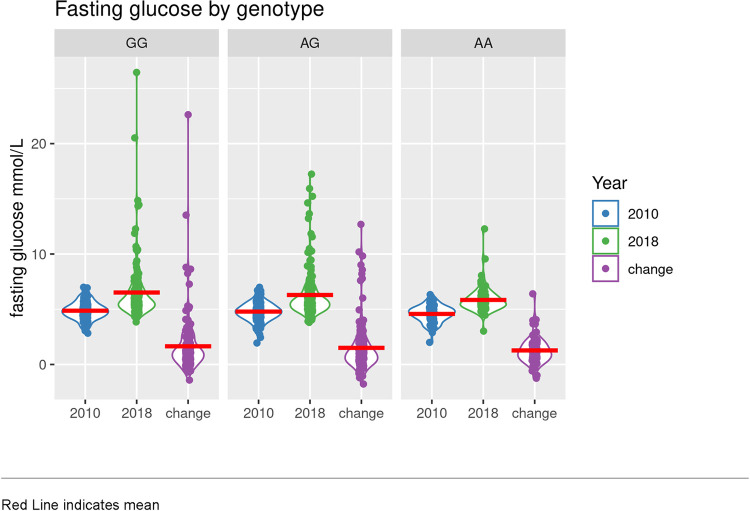
Fasting glucose (mmol/L) by genotype in 2010 (blue) and 2018 (green), and change in FG (purple), in *n* = 401 adult Samoans who did not have diabetes in 2010 or were not using diabetes medications at either timepoint. Red line indicates the mean.

Table 2 shows participant characteristics in 2010 and 2018 by genotype. By 2018, 21.6% individuals with the GG genotype; 17.9% individuals with AG genotype, and 9.1% individuals with AA genotype developed type 2 diabetes mellitus (type 2 diabetes mellitus prevalence in 2018 by genotype group, *p* = 0.06). Average FG levels increased in all genotype groups between 2010 and 2018: mean change in FG was 1.6 ± 2.5 mmol/L among those with the GG genotype; 1.5 ± 2.2 mmol/L among those with the AG genotype, and 1.3 ± 1.2 mmol/L among those with the AA genotype (Fig 2). While mean change in FG between 2010 and 2018 was lowest among those with the AA genotype, differences between the genotype groups did not meet the threshold for statistical significance (*p* = 0.22). Between 2010 and 2018, BMI increased among all genotypes: 1.9 ± 3.2 kg/m^2^ among those with the GG genotype; 2.1 ± 3.6 kg/m^2^ among those with the AG genotype; 3.3 ± 4.0 kg/m^2^ among those with the AA genotype (*p* = 0.01).

**Table 2:** Cohort characteristics stratified by rs373863828 genotype and time point (*n* = 401).

	GG (*n* = 162)			AG (*n* = 162)			AA (*n* = 77)		
	2010	2018	*p* values	2010	2018	*p* values	2010	2018	*p* values
Age (years)	43.0 (10.1)	51.2 (10.1)	-	43.5 (10.0)	51.8 (10.0)	-	41.4 (10.1)	49.5 (10.1)	-
BMI (kg/m^2^)^a^	32.6 (6.1)	34.5 (6.7)	3.0 × 10^−12^	34.5 (6.5)	36.6 (7.9)	5.0 × 10^−12^	35.1 (7.6)	38.4 (10.1)	6.8 × 10^−10^
BMI change (kg/m^2^)^a^	1.9 (3.2)			2.1 (3.6)			3.3 (4.0)		
BMI rate-of-change ((kg/m^2^)/year)	0.23 (0.38)			0.26 (0.44)			0.40 (0.50)		
Male (%)	77 (47.5%)	-	-	76 (46.9%)	-	-	35 (45.5%)	-	-
Current Smoker (%)	63 (38.9%)	60 (37.0%)	0.677	63 (38.9%)	71 (43.8%)	0.170	36 (46.8%)	32 (41.6%)	0.423
Meets PA recommendations (%)	53 (32.7%)	44 (27.2%)	0.313	49 (30.2%)	35 (21.6%)	0.082	26 (33.8%)	26 (33.8%)	1.000
Household asset score	5.4 (2.9)	6.5 (2.9)	9.4 × 10^−6^	5.8 (2.6)	6.6 (2.8)	2.4 × 10^−4^	5.8 (3.1)	6.7 (2.9)	0.007
***Metabolic phenotypes***									
Type 2 Diabetes (%)^b^	0	35 (21.6%)	9.1 × 10^−9^	0	29 (17.9%)	2.0 × 10^−7^	0	7 (9.1%)	0.023
Average Type 2 Diabetes Incidence/year^c^	0	4.4	CI: 3.0–6.1	0	3.6	CI: 2.4–5.2	0	0.9	CI: 0.4–1.8
Fasting glucose (mmol/L)	4.9 (0.8)	6.5 (2.7)	3.6 × 10^−14^	4.8 (0.9)	6.3 (2.2)	2.2 × 10^−15^	4.6 (0.8)	5.8 (1.2)	3.3 × 10^−14^
Fasting glucose change (mmol/L) 2010–2018 ^c^	1.6 (2.5)			1.5 (2.2)			1.3 (1.2)		
Fasting glucose rate-of-change ((mmol/L)/year)^d^	0.20 (0.31)			0.18 (0.27)			0.16 (0.15)		
***Census Region* (*%*)**									
Apia Urban Area (AUA)	37 (22.8%)	37 (22.8%)	1.000	25 (15.4%)	29 (17.9%)	0.221	23 (29.9%)	22 (28.6%)	1.000
Northwest ‘Upolu (NWU)	71 (43.8%)	69 (42.6%)	0.683	70 (43.2%)	67 (41.4%)	0.371	28 (36.4%)	28 (36.4%)	1.000
Rest of ‘Upolu (ROU)	54 (33.3%)	56 (34.6%)	0.683	67 (41.4%)	66 (40.7%)	1.000	26 (33.8%)	27 (35.1%)	1.000

mean (SD) or *n* (%)

*p* values for difference in values between 2010 and 2018 calculated with Student’s paired *t* test for continuous traits and McNemar’s test for binary traits.

^a^ BMI change by 2018 by genotype, *p* = 0.01

^b^2010 Type 2 diabetes prevalence of 0 is due to the selective sampling strategy of excluding those with type 2 diabetes.

^c^ 2018 prevalence of type 2 diabetes by genotype, *p* = 0.06

^d^ p value for difference in FG change between genotypes calculated with ANOVA, *p* = 0.22

^e^*p* value for difference in FG rate-of-change between genotypes calculated with ANOVA; *p* = 0.25

CI indicates the 95% confidence interval for the mean incidence of T2D per year of the Poisson distribution using the exact method.

### FG rate-of-change

**Fig 3:**
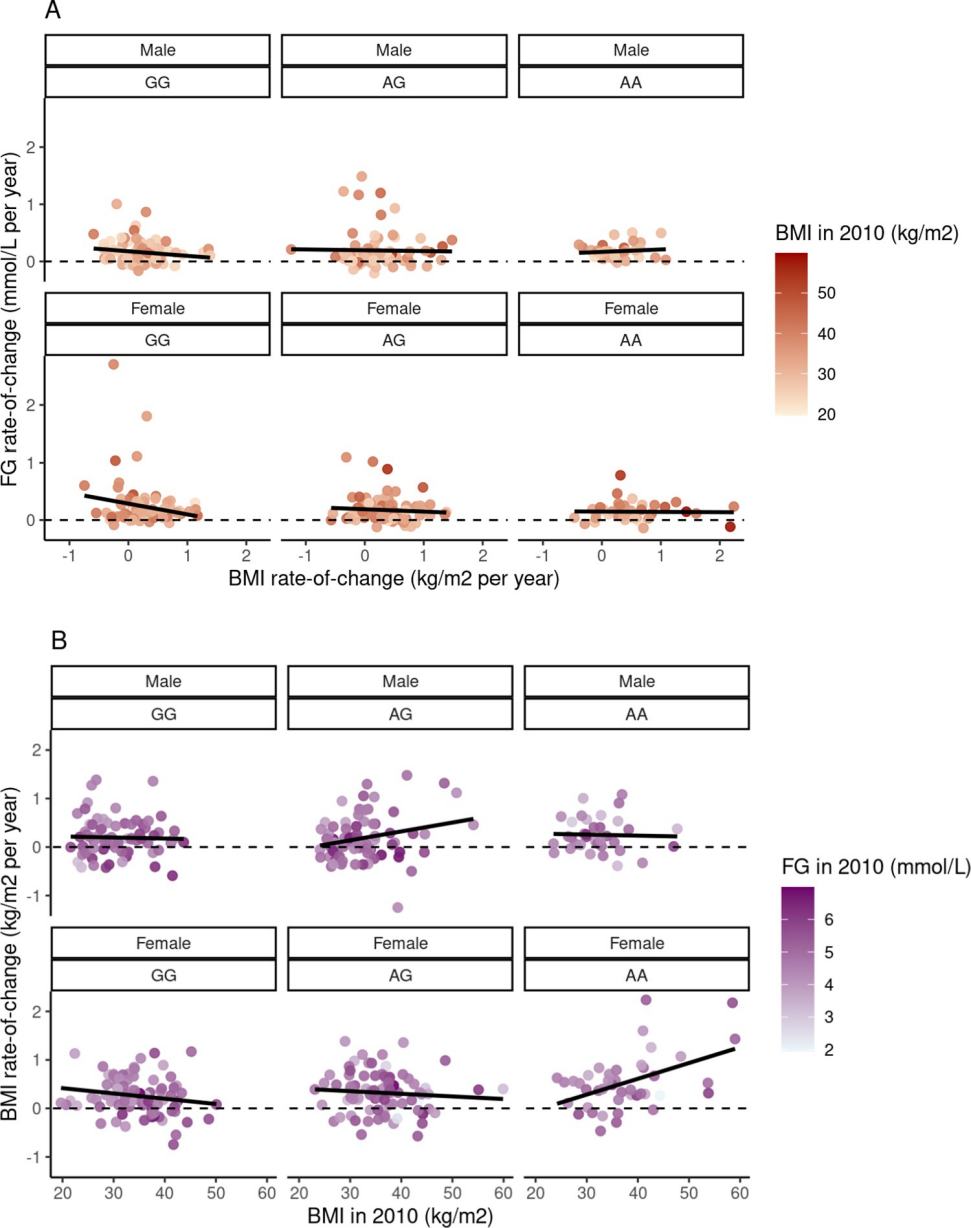
**(A)** Fasting glucose rate-of-change (mmol/L per year) by BMI rate-of-change (kg/m^2^ per year) stratified by sex and genotype. The color of points indicates baseline BMI. Dashed line represents no change in fasting glucose. Solid line is a superimposed linear trend line. **(B)** BMI rate-of-change (kg/m^2^ per year) by baseline BMI (kg/m^2^) stratified by sex and genotype. The color of points indicates baseline fasting glucose values. Dashed line represents no change in BMI. Solid line is a superimposed linear trend line.

In the multivariable analysis of FG rate-of-change (change in FG per year), baseline BMI was associated with higher FG rate-of-change among men and women (β = 0.01 mmol/L/year per kg/m^2^, *p* = 2.0 x 10^−6^ in total sample) (Table 3). BMI rate-of-change between 2010 and 2018 was negatively associated with FG-rate-of-change, indicating that participants with higher BMI rate-of-change had a lower FG rate-of-change (Table 3 and Fig 3A).

**Table 3:** Associations between cohort characteristics and fasting glucose rate-of-change/year between 2010 and 2018 from multivariable model (*n* = 401).

	Combined (*n* = 401)		Men (*n* = 188)		Women (*n* = 213)	
**Variables**	**Estimate (SE)**	***p* value**	**Estimate (SE)**	***p* value**	**Estimate (SE)**	***p* value**
Intercept	−0.03 (0.24)	-	−0.25 (0.34)	-	0.05 (0.37)	-
2010 Age (years)	0.01 (0.01)	0.496^a^	0.01 (0.02)	0.487^†^	−0.001 (0.02)	0.776^†^
2010 Age^2^	−7.3 × 10^−5^ (1.2 × 10^−4^)		−1.8 × 10^−4^ (1.7 × 10^−4^)		−6.8 × 10^−6^ (1.9 × 10^−4^)	
Sex (ref: male)	−0.02 (0.03)	0.444	-	-	-	-
2010 BMI (kg/m^2^)	0.01 (0.002)	2.0 × 10^−6^	0.01 (0.003)	0.005	0.01 (0.003)	2.9 × 10^−4^
BMI rate-of-change (kg/m^2^)/year	−0.09 (0.03)	0.004	−0.07 (0.05)	0.140	−0.11 (0.05)	0.022
2010 Fasting glucose (mmol/L)	−0.04 (0.02)	0.023	−0.04 (0.02)	0.094	−0.04 (0.03)	0.178
rs373863828 genotype ^b^	−0.03 (0.02)	0.068	−0.004 (0.03)	0.863	−0.05 (0.03)	0.058
2010 NWU census region (ref: AUA)	0.04 (0.04)	0.226	0.02 (0.05)	0.665	0.06 (0.05)	0.226
2010 ROU census region (ref: AUA)	0.02 (0.04)	0.613	0.003 (0.05)	0.946	0.03 (0.05)	0.592
2010 Meets PA recs (ref: no)	0.03 (0.03)	0.360	0.02 (0.04)	0.575	0.03 (0.04)	0.562
2010 Household asset (scores)	−0.001 (0.005)	0.841	0.004 (0.01)	0.586	−0.01 (0.01)	0.494
2010 Smoking (ref: no)	0.02 (0.03)	0.562	0.01 (0.04)	0.817	0.04 (0.04)	0.414

AUA: Apia Urban Area, NWU: Northwest ‘Upolu, ROU: Rest of ‘Upolu; Meets PA recs: meets WHO recommendations for weekly MET minutes. Individuals taking diabetes medications at either timepoint were excluded.

^a^ Calculated with a partial *F* test that compared a model with age and age^2^ to one without either term.

^b^ Parameter estimates are per copy of the A allele of rs373863828

Baseline FG was also negatively associated with FG rate-of-change in the total sample (β = −0.04 mmol/L per year, *p =* 0.023).

The estimated effect of the A allele of the rs373863828 genotype on FG rate-of-change was negative in the total sample (β = −0.03 mmol/L/year per allele, *p =* 0.068) indicating the A allele was associated with lower FG rate-of-change, after accounting for baseline FG, baseline BMI, and BMI rate-of-change, among other covariates. The magnitude of effect of the rs373864828 genotype was larger in women, compared to men in sex-stratified analyses (β = -0.004 mmol/L/year per allele, *p* = 0.863 in men, β = -0.05 mmol/L/year per allele, *p* = 0.068 in women).

We observed that there does not appear to be a strong relationship between the A allele, BMI rate-of-change, and FG rate-of-change (Fig 3A). The negative relationship between BMI rate-of-change and FG rate-of-change (β = -0.09 mmol/L/year per kg/m^2^/year, *p* = 0.004; Table 3), appears to be driven by those with the GG genotype. Additionally, there does not appear to be a strong overall relationship between the A allele, baseline BMI, and BMI rate-of-change, a despite the strong, positive association between baseline BMI and BMI rate-of-change observed among males with the AG genotype and females with the AA genotype (Fig 3B).

## Discussion

The aim of this study was to (1) describe the development of type 2 diabetes mellitus and changes in fasting glucose (FG) between 2010 and 2018 among a longitudinal cohort of adult Samoans without type 2 diabetes mellitus or who were not using diabetes medications at baseline, and (2) to examine associations between FG rate-of-change (mmol/L per year) and the A allele of rs373863828. Between 2010 and 2018, type 2 diabetes mellitus developed among 17.7% of the sample, inclusive of both sexes and among all genotype groups. As expected, the substantial percentage of type 2 diabetes mellitus cases was concomitant with increases in both FG and BMI. The average annual rate of change in FG was, however, associated with several 2010 characteristics, including rs373863828 genotype.

In line with our previous study of these data, the rs373863828 A allele was associated with greater increases in BMI over time [6, 30]. In this analysis, higher BMI rate-of-change was associated with an *attenuated* increase in FG (i.e., FG rate-of-change closer to 0), although this relationship appeared to depend upon genotype. Among AG and AA genotype individuals, there did not appear to be a strong relationship between BMI rate-of-change and FG rate-of-change; individuals in these groups had, on average, a small increase in FG over time that was similar regardless of a person’s BMI rate-of-change during the same period. However, for those with the GG genotype, a higher BMI rate-of-change appeared to be connected to a lower FG rate-of-change. It is therefore possible that the rs373863828 genotype moderates the relationship between BMI rate-of-change and FG rate-of-change, although this study did not test this moderation formally due to small sample size. Notably, it is also possible that this relationship may also be reflecting age-, and/or hyperglycemia and insulin deficiency-related weight loss (i.e., muscle atrophy) [30–33].

Also as expected, the A allele was associated with higher baseline BMI [6] but lower baseline FG. We observed that the rs373863828 genotype appears to moderate the relationship between baseline BMI and increase in BMI. Among women with the AA genotype, baseline BMI was associated with a greater increase in BMI overall, compared to the AG and GG genotype individuals, as we saw in our previous study of these data [30].

These results highlight the complexity of the effects of rs373863828 and add to the body of evidence for the paradoxical association of the A allele of rs373863828 with higher BMI, yet simultaneously lower FG. Here we demonstrated that even after accounting for baseline FG and both baseline BMI and BMI rate-of-change, the A allele of rs373863828 may have an attenuating effect on FG levels over time (i.e., conferring an additional protective effect against the development of type 2 diabetes mellitus), particularly apparent in women. As observed, those with the AA genotype were less likely to develop type 2 diabetes mellitus by 2018 compared to their GG and AG peers (even though those with the AA genotype experienced a larger increase in BMI during that same period). This notable finding may be due to a direct effect of rs373863828 impacting the biological mechanisms underlying type 2 diabetes mellitus (e.g., beta cells) above and beyond its effect on body composition [34].

However, the mechanisms by which the A allele of rs373863828 improves glycemia and reduces risk of type 2 diabetes mellitus remain unknown. Glycemia may be influenced by insulin- and non-insulin-mediated mechanisms. The preponderance of evidence related to genetic contributions to diabetes mellitus suggests that most “diabetes-risk” loci influence beta cell function [35, 36]. In contrast, most “obesity-risk” loci influence CNS pathways regulating energy intake and expenditure [37–40]. A recent study from Burden et al. [41] showed that male Māori carriers of the A allele had increased insulin secretion without changes in peripheral insulin sensitivity (females were not studied) [41]. These data suggest the A allele may act by increasing beta cell mass and/or function, leading to improved insulin secretion. However, a recent pig model with the orthologous A allele reported improved glycemia secondary to adipocyte hyperplasia, adipose tissue expandability, and improve peripheral insulin sensitivity [42]. Other possibilities by which the A allele could improve FG or reduce type 2 diabetes mellitus risk include, but are not limited to, reducing hepatic gluconeogenesis, improving non-insulin mediated glucose disposal (i.e., by muscle), reducing carbohydrate absorption, enhancing glycosuria, etc. Additional studies in humans and/or preclinical models are required to further characterize the physiological and molecular mechanisms underlying these effects. Our findings also suggest other interesting directions for future research. For example, we do not understand why the effect appears to be stronger in women compared to men. We also do not know if, among the carriers of the A allele of rs373863828, the pace of type 2 diabetes mellitus progression may be slower or if these individuals have reduced risk of hyperglycemia-related complications [43]. Accordingly, we advocate for further examination of how the allele interacts with type 2 diabetes mellitus pathogenesis and progression.

High BMI is a known, independent, modifiable, and major risk factor for type 2 diabetes mellitus [44–46]. Our results suggest that high BMI, and weight gain over time contributed to increasing FG and the development of diabetes mellitus as found in other populations [44–47]. While the impact of these risk factors appears to be somewhat attenuated in those with AA and AG genotypes, we observed increasing FG among the whole cohort and a substantial proportion of participants developing type 2 diabetes mellitus. We also observed increases in other known risk factors for type 2 diabetes mellitus—impacting the sample as a whole—including improvements in socioeconomic position, based on ownership of household assets (findings that are in line with ongoing economic development and associated increases in non-communicable disease prevalence documented in Samoa [16] and the Pacific region more broadly) [3, 47–50], and declines in physical activity levels among the sample, and especially among women. There is, consequently, a need to reduce modifiable risk factors among all adults and enhance diabetes prevention programming in this setting.

### Strengths and weaknesses

Our study provides the first longitudinal exploration of the associations between the A allele of rs373863828, FG rate-of-change, and type 2 diabetes. The study has provided insight into the factors influencing FG increase in Samoan adults but is limited in several ways. Our analyses do not consider dietary intake. Additionally, we cannot readily explain the differences we observed between the sexes, increases in FG, and the A allele of rs373863828. It is possible that the differences may be an artifact of our relatively small sample size, and/or representativeness of the sample; however, they could also speak to larger sex differences in the expression and impact of the A allele of rs373863828 on FG. The sampling strategies utilized here (i.e., the removal of individuals using diabetes medications and those with diabetes in 2010) led to an analytic sample with higher BMI, younger ages, and a higher frequency of individuals who smoke and met physical activity recommendations (compared to those excluded); additionally, the over-representation of individuals with AG and AA genotypes (based on the 2018 approach to recruitment) make the findings non-representative for the larger Samoan population (or of the 2010 GWAS sample). Furthermore, our sampling strategy (excluding those with diabetes in 2010 and including individuals with diabetes in 2018) may have biased the estimation of mean FG levels at both timepoints. Survivor bias may also be impacting the findings observed here. It is also likely that our results are complicated by the strong association between genotype and BMI [6]. We also cannot be sure whether participants, if they had been told that they had elevated FG in 2010 (e.g., within the range of prediabetes), may have purposively tried to lose weight. While this was partially controlled for by not enrolling individuals in 2017–2019 who experienced greater than 5% weight loss within the 12-months preceding study enrollment (Fig 1), weight control activities for the years prior to then remain unknown. Along with age-, and hyperglycemia and insulin-deficiency-related weight loss, these potential factors could be influencing the associations between BMI rate-of-change and FG rate-of-change observed here. We advocate for future studies to conduct similar analyses with larger sample sizes, additional measures of glycemia, insulin resistance and insulin sensitivity, and increased number of data collection time points to better understand the complex relationship between the rs373863828 genotype and type 2 diabetes mellitus.

## Conclusions

In summary, our study provides the first longitudinal assessment of associations between increases in FG over time and the A allele of rs373863828. While we demonstrate preliminary support for a potential relationship between lower FG rate-of-change and the A allele of rs373863828, the effect is not readily observed in men, and may have been overshadowed by increases in other type 2 diabetes mellitus risk factors among many study participants. Further study is needed to explore and validate these findings. The development of type 2 diabetes mellitus among a substantial portion of the sample and the observed increases in other known diabetes-associated risk factors, calls for the continued promotion of diabetes prevention programming and modifiable risk factor reduction in Samoa.

## Supporting information

S1 Text Samoan translation of abstract. (DOCX)
