# Supplementary material for: Associations between fasting glucose rate-of-change and the missense variant, rs373863828, in an adult Samoan cohort
Source: PLoS One. 2024 Jun 3;19(6):e0302643. doi: 10.1371/journal.pone.0302643 (PMC11146712; doi:10.1371/journal.pone.0302643)
Supplement: S1 Text — (DOCX) [file pone.0302643.s001.docx]

Aotelega

Manulauti

O manulauti, (1) o faamatalaga i le gasologa o le gasegase suka i lona vaega 2 ma suiga i le suka ao lei tausami (FG) i le va o le 2010 agai i le 2018, e iai tagata Samoa e lei aafia i le gasegase suka vaega lona 2, e lei faaaogaina foi ni togafitiga sa faatinoina ai le suesuega, ma le vaega (2), sailia fesootaiga i le va o le suka ao lei tausami (FG) ma le saoasaoa o suiga i le tausaga ma le *A allele of rs373863828 in CREBRF,* lea e itiiti se vaega e aafia ai i le ituaiga 2 o le suka.

Metotia

E talalasi ma mau eseese i le sailiga o le suka ae lei tausami (FG), i le faagasologa o le vaega 2 o le gasegase suka, ga’o o le tino (DMI), o tausaga o le soifua, taumafa tapaa, gaioiga, nofoaga tu lata i le taulaga, o meafale e faaaogaina e tagata i aso uma i mea e manaomia ai i le va o le 2010 i le 2018, e to’a 401 tagata matutua i Samoa sa faaaogaina o lenei suesuega, o i latou na filifilia, e iai le *~2:2:1 ratio of GG:AG:AA rs373863828* o ituaiga sela o le tino. E tasi a le fua faatatau o le suka sa faaaogaina e sailia ai pe e iai se sootaga o le suka ae lei tausami (FG) ma le *rs373863828* o ituaiga sela o le tino, ituaiga tagata, fua faatatau o le suka ae lei tausami (FG), pe a fua agai i le matua o le soifua, nofoaga, ga’o o le tino, taumafa tapaa, gaioga, meafale faaaogaina i aso uma, faapea ai ma le suiga o le ga’o o le tino (BMI). O sui auai uma na aafia i le vaega 2 o le suka pe sa faaaogaina foi ni togafitiga o le suka i le 2010, sa le mafai ona auai i lenei sailiiliga.

Aotelega

I le tausaga e 2018, na aliae ai le vaega lona 2 o le suka, ia i latou na faatinoina ai le suesuega, 20.2% mai le ituaiga o tamaloloa ma le 15.5% mai le ituaiga o fafine ma o le maualuga faatulagaina o le vaega lona 2 o le suka, e eseese lava, fua i le ituaiga sela o le tino *rs373863828,* i vaega eseese nei *(21.6% among GG; 17.9% among AG; 9.1% among AA: p = 0.06).* E iai foi le suiga i le ga’o o le tino ma le suka ae lei tausami iai latou uma sa faatino ai le sailiiliga mai le 2010 agai le 2018. O le fua faatatau sa faaaogaina, ua iloa ai se vaega itiiti o le *A allele* sa fesoasoani i le suiga o le suka ao lei tausami (FG) i lea piriota *(β = −0.05 mmol/L/year/allele, p = 0.058 among women; β = −0.004 mmol/L/year/allele, p = 0.863 among men),* pe a fua i le vaega na amata mai ai, faatasi ai ma isi vaega moomia ma le suiga i le ga’o o le tino.

Faaiuga

Na maitauina le maualuga o le tulaga i le suka ao lei tausami (FG) faatasi ai ma le tulaga i le gao o tino (BMI) i totonu o le valu tausaga, e mafua mai i le maualuga o le lemū o le fua faatatau o le suka ao lei tausami (FG), i tina oloo iai le ituaiga sela o le AG ma le AA. I sona faaopoopoga, o se fuainumera o tagata taitoatasi na amatalia ai le vaega 2 o le gasegase o le suka i le tausaga e 2018 (pito sili ona maualuga i le ituaiga sela o le GG). E manaomia le faalauaitele o suesuega ina ia malamalama ai i le aafia o le *A allele* mai i le suka ao lei tausami(FG) ma le tuputupuae o le vaega 2 o le gasegase o le suka ma iloilo ai pe faapefea e le ga’o o le tino(BMI) ma le eseese o ituaiga tagata ona aafia ai lea fesootaiga. A fua ia matou suesuega i le faatuputupulaia o tulaga ogaoga i lea taimi ma le faagasologa o le vaega 2 o le gasegase o le suka ia i latou na auai, matou te fautuaina ai le faaauauina pea o faalauiloa mo puipuiga ma polokalame mo le togafitiga o le gasegase o le suka ma ia faaitiitia ai le tele o le aafiaga i lea vaega.
